# An Essential Function for the ATR-Activation-Domain (AAD) of TopBP1 in Mouse Development and Cellular Senescence

**DOI:** 10.1371/journal.pgen.1003702

**Published:** 2013-08-08

**Authors:** Zhong-Wei Zhou, Cong Liu, Tang-Liang Li, Christopher Bruhn, Anja Krueger, WooKee Min, Zhao-Qi Wang, Antony M. Carr

**Affiliations:** 1 Leibniz Institute for Age Research – Fritz Lipmann Institute (FLI), Jena, Germany; 2 Sussex for Genome Damage and Stability Centre, School of Life Sciences, University of Sussex, Brighton, Sussex, United Kingdom; 3 Faculty of Biology and Pharmacy, Friedrich-Schiller-University Jena, Jena, Germany; University of Washington, United States of America

## Abstract

ATR activation is dependent on temporal and spatial interactions with partner proteins. In the budding yeast model, three proteins – Dpb11^TopBP1^, Ddc1^Rad9^ and Dna2 - all interact with and activate Mec1^ATR^. Each contains an ATR activation domain (ADD) that interacts directly with the Mec1^ATR^:Ddc2^ATRIP^ complex. Any of the Dpb11^TopBP1^, Ddc1^Rad9^ or Dna2 ADDs is sufficient to activate Mec1^ATR^ *in vitro*. All three can also independently activate Mec1^ATR^ *in vivo*: the checkpoint is lost only when all three AADs are absent. In metazoans, only TopBP1 has been identified as a direct ATR activator. Depletion-replacement approaches suggest the TopBP1-AAD is both sufficient and necessary for ATR activation. The physiological function of the TopBP1 AAD is, however, unknown. We created a knock-in point mutation (W1147R) that ablates mouse TopBP1-AAD function. TopBP1-W1147R is early embryonic lethal. To analyse TopBP1-W1147R cellular function *in vivo*, we silenced the wild type TopBP1 allele in heterozygous MEFs. AAD inactivation impaired cell proliferation, promoted premature senescence and compromised Chk1 signalling following UV irradiation. We also show enforced TopBP1 dimerization promotes ATR-dependent Chk1 phosphorylation. Our data suggest that, unlike the yeast models, the TopBP1-AAD is the major activator of ATR, sustaining cell proliferation and embryonic development.

## Author Summary

DNA damage checkpoint signalling is an essential component of the DNA damage response. Many of the key proteins initiating the checkpoint signal have been identified and characterised in yeast. Here we explore the role of the ATR activating domain (AAD) of TopBP1 in embryonic development, cell growth and checkpoint activation using a mouse model. In contrast to yeasts, where the TopBP1 AAD plays a redundant, and thus phenotypically minor, role in ATR activation, our data demonstrate that the mouse TopBP1 AAD is essential for cellular proliferation. Interestingly, this suggests evolution has provided a simpler ATR activation mechanism in metazoans than it has in yeasts.

## Introduction

In response to endogenous and exogenous stress, cells have evolved a range DNA damage response (DDR) pathways to maintain genomic stability [1], [2], [3], [4]. In all eukaryotes, two evolutionarily conserved PI3-kinase-like protein kinases, ATM (Ataxia telangiectasia mutated) and ATR (ATM- and Rad3-related) respond directly to DNA damage to control cell-cycle progression and regulate other DNA damage responses such as DNA repair and apoptosis. ATM activation is triggered by double-strand breaks (DSBs), whereas ATR activation is induced by single stranded DNA (ssDNA) occurring due to replication stress, resected-DSBs or other single strand lesions [5], [6], [7], [8].

In all eukaryotic organisms, ATR is found associated with ATRIP (ATR-interacting protein) which is necessary to recruit ATR to RPA-coated ssDNA [9], [10], [11], [12]. In metazoans, but not in yeasts, this correlates with ATR autophosphorylation at T1989 [13], [14]. Pre-requisite for ATR activation is the independent recruitment of the Rad17-RFC checkpoint clamp loader to the junction of RPA-coated ssDNA and double stranded DNA (dsDNA), where it facilitates the loading of the Rad9-Hus1-Rad1 (9-1-1) sliding clamp [15]. The co-recruitment of ATR and the 9-1-1 clamp establishes a platform for activation of the ATR pathway [13], [16], [17]. The C-terminus of the Rad9 subunit of the 9-1-1 clamp is responsible for recruiting TopBP1 [18], [19], [20], a conserved multi-BRCT-domain scaffolding protein [21]. In yeast model systems, the Rad9 C-terminus must be phosphorylated by ATR to provide a docking site for phospho-protein binding domains within TopBP1 [18], [20]. In metazoans, Rad9 is constitutively phosphorylated by CK2 and thus TopBP1 recruitment does not require ATR-dependent Rad9 phosphorylation.

Metazoan TopBP1 contains nine BRCA1 C-terminal (BRCT) domains while the yeast homologs contain only four BRCT domains. The TopBP1 BRCT domains define phospho-binding motifs [22], [23] that allow TopBP1 to scaffold distinct proteins and protein complexes in response to the phosphorylation status of its clients. In all eukaryotes, TopBP1 plays an essential role in the initiation of DNA replication [21]. In yeast models, this function reacts to cell cycle-dependent phosphorylation of two client proteins, Sld2 and Sld3, allowing TopBP1 to bridge an interaction between two replication factors in order to promote Cdc45 and GINS loading to activate the replicative helicase [24]. A similar role is evident in metazoans, where TopBP1 association with the Sld2 homolog, Treslin, is essential for replication initiation [25], [26], [27].

In response to ssDNA during DNA replication stress or DNA repair, yeast TopBP1 performs an equivalent scaffolding role, bridging between the 9-1-1 checkpoint clamp and the checkpoint mediator proteins (the 53BP1 homologs) which present checkpoint effector kinases (Chk's) to ATR [28], [29], [30]. This scaffolding role is essential for a functional ATR-Chk response. In addition, yeast TopBP1 contains a conserved ATR activation domain (AAD) which, when over-expressed, can directly induce ATR activation in the absence of DNA damage 11,31,32. The yeast TopBP1 AAD participates in, but is not necessary for, ATR activation: AAD-deficient separation of function mutants display only sensitivity to genotoxins and partial, cell cycle-specific checkpoint defects [32], [33]. As observed in the yeast models, metazoan TopBP1 is similarly required for activation of the ATR-Chk1 axis and is recruited to the site of DNA damage by binding to the C-terminus of the 9-1-1 complex [16], [31], [34]. However, at this point, significant differences emerge between the yeast and metazoan systems: in addition to the constitutive formation of a 9-1-1 TopBP1 interaction in metazoans (see above), replacing Xenopus TopBP1 with a recombinant protein containing a mutation in the AAD (W1138) completely blocks ATR activation in response to replication inhibition in extracts [31]. While extracts may not fully recapitulate all aspects of the cellular environment, this suggests a more important role for the metazoan TopBP1 AAD in ATR activation when compared to yeast.

The differences between ATR activation in the yeast and the metazoan systems are intriguing. In the yeast models, ATR provides the bulk of the checkpoint signalling following all forms of DNA damage, including DSBs. In metazoans, ATM provides the majority of checkpoint signalling in response to DSBs, with ATR playing a minor role. This distinction between yeasts and metazoans can be explained, at least in part, by different repair priorities: yeasts generally rapidly resect DSBs for repair by homologous recombination, with non-homologous end joining (NHEJ) - which occurs without significant resection - playing a minor role. Conversely, metazoan cells rely largely on NHEJ and thus detect DSBs through the ATM pathway. Experimentally limiting resection in yeast models uncovers an ATM-dependent checkpoint [35], [36], demonstrating the underlying machinery is conserved. The distinct repair priorities between yeast and metazoan systems are likely to underpin changes in the architecture of ATR activation mechanisms during evolution. For example, it is notable that distinct pairs of BRCT-domains mediate the 9-1-1 interaction in yeasts and metazoans: 9-1-1 interacts with BRCT 3+4, in yeasts (homologous to metazoan BRCT 4+5) but with the conserved BRCT1+2 pair in metazoans [21]. Furthermore, BRCT domains 7/8 in metazoans (which is not conserved in yeasts) binds autophosphorylated ATR-T1989 to promote a tight complex and strengthen ATR signalling [13], [14].

Complete deletion of TopBP1 in untransformed mouse or human primary cells induces cellular apoptosis and TopBP1 deficiency results in an early embryonic lethality [37], [38], [39], [40], [41]. Tissue specific deletion of TopBP1 in the central nervous system (CNS) similarly leads to an accumulation of DNA breaks in neuronal progenitors and subsequent disruption of neurogenesis [42]. These data are consistent with the essential role for TopBP1 in the initiation of DNA replication. To specifically establish the physiological function of the TopBP1 AAD, and to investigate if it is dispensable for ATR activation in metazoans as it is in yeasts, we generated a mouse model with a specific knock-in AAD mutation. We show that the TopBP1 AAD is essential for the embryonic development, phenocopies the lethal phenotype of ATR and is necessary for ATR signalling after UV damage. These data strongly suggest that, unlike in the yeast models, ATR activation of by the TopBP1 AAD is the major, if not only, route to activating the ATR-Chk1 axis and is essential for cell proliferation and survival.

## Results

**Figure 1:**
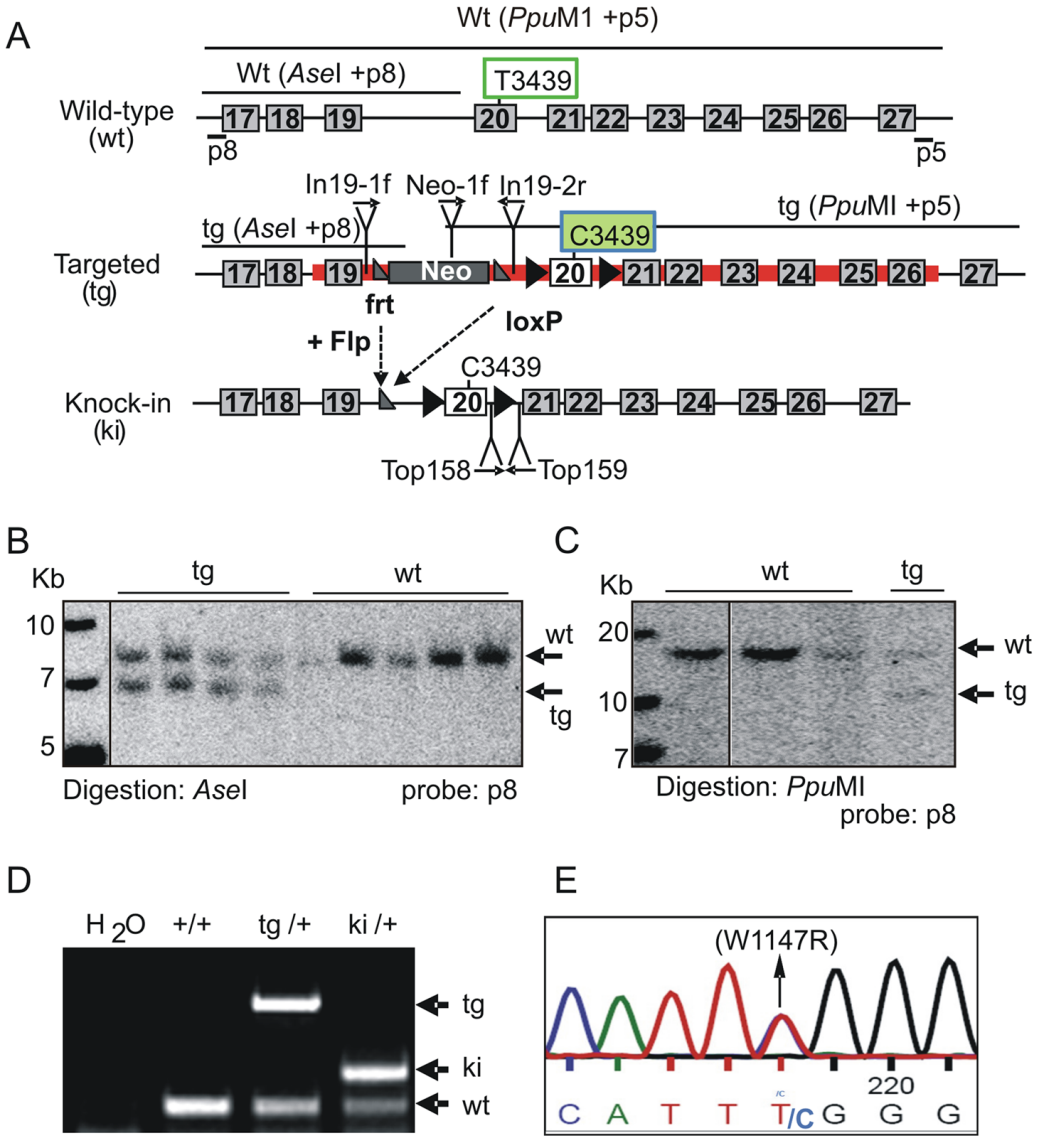
Generation of TopBP1 AAD mutant transgenic mice. (**A**) Schematic of the C-terminus of the *TopBP1* locus: wild type (wt), targeted (tg) and knock-in (ki) alleles. The red line marks the targeting vector. Exons are numbered in the boxes, Southern blot probes (p8 and p5), sizes of DNA fragments after indicated enzyme digestion and the location of primers for PCR genotyping are shown. The targeting vector contained a neomycin resistance gene (Neo) flanked by two *frt* sites (grey triangles). Exon 20 is flanked by two *loxP* sites (black triangle). Tryptophan 1147 (W1147) was mutated into arginine in AAD by replacing T3439 with C in exon 20. (**B–C**) Southern blot analyses of gene targeted ES cell clones: Homologous integration was verified by digestion with *Ase*I and hybridization with a 5′ external probe (p8) and by *Ppu*MI digestion and hybridization with a 3′ external probe (p5). (**D**) PCR genotyping analysis of the wild type, targeted and the knock-in allele following Neo excission. (**E**). Sequencing of genomic DNA from *TopBP1^ki/+^* heterozygous MEFs confirms the mutation of TTT (tryptophan) to TTC (Arginine) (W1147R).

To explore the function of the TopBP1 AAD, we generated a specific knock-in allele of TopBP1 by gene targeting in mouse ES cells. The AAD domain spans exons 19 and 20 (Fig. S1), with the core indispensable aromatic residue, W1147 [31] encoded within exon 20. To change W1147 to arginine (W1147R), T3439 was mutated to C3439 in a targeting vector (Fig. 1A). A neomycin resistance cassette (Neo) flanked by Frt sequences, was also inserted into intron 19. Following electroporation into ES cells, 9/200 neomycin-resistant clones contained the targeted allele (Fig. 1B,C). A correctly targeted ES clone was used to derive germline chimeric mice (designated as *TopBP1* ^tg/+^). Crossing *TopBP1* ^tg/+^ mice with Flp transgenic mice (Jackson laboratory strain 003946) led to generation of the desired TopBP1 AAD knock-in allele, designated *TopBP1* ^ki/+^ (Fig. 1A,D). The point mutation was confirmed by sequencing mouse genomic *TopBP1* ^ki/+^ DNA (Fig. 1E) and verified in cDNA from *TopBP1* ^ki/+^ MEFs (Fig. S2A). As expected, TopBP1 protein levels were similar between *TopBP1* ^+/+^ and *TopBP1* ^ki/+^ MEFs (Fig. S2B). We also confirmed that the AAD mutation did not compromise the protein expression of TopBP1 when transfected into Cos7 cells (Fig. S2C).

### Inactivation of TopBP1 AAD results in early embryonic lethality

**Figure 2:**
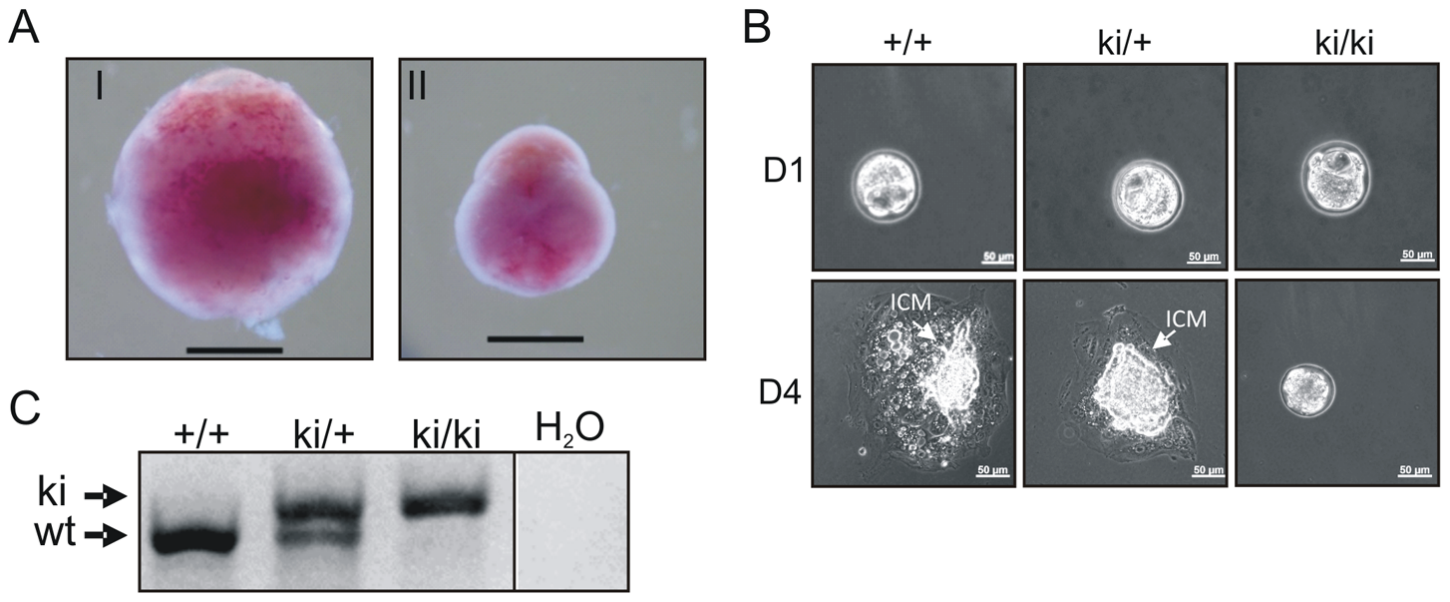
Inactivation of the AAD results in early embryonic developmental defects. (**A**) Decidua at E11.5 from intercrossing of *TopBP1^ki/+^*mice. Decidua in (I) was genotype ki/+. Decidua in (II) was empty, thus with unclear genotype. Bar = 1 mm. (**B**) Cultures of E3.5 blastocysts from *TopBP1^ki/+^* intercrosses. D1: 1 day after culture, D4: 4 days after culture. Arrows indicate inner cell mass (ICM). Bar = 50 micrometers. Genotypes are indicated on the top of respective images. (**C**) Example of PCR genotyping from ICM of blastocyst outgrowth. Expected product size for alleles labeled. ki = knock-in; wt = wild type.

Heterozygous *TopBP1* ^ki/+^ mice are viable and phenotypically normal during a 2 year-observation period (data not shown). However, no homozygous *TopBP1* ^ki/ki^ offspring were obtained from *TopBP1* ^ki/+^ intercrosses (167 live births genotyped, Table 1). Backcrossing *TopBP1* ^ki/+^ with *TopBP1* ^+/+^ gave the expected ratio of *TopBP1* ^ki/+^ and *TopBP1* ^+/+^ offspring, indicating that there were no fertility defects in either the male or female *TopBP1* ^ki/+^ animals (Table 1). We thus analyzed deciduas and embryos derived from *TopBP1* ^ki/+^ intercrosses: genotyping at E11.5 revealed no homozygous mutants (Table 1), although 17/48 deciduas were small, precluding reliable PCR due to the presence of mother-derived tissues (Fig. 2A). We next isolated blastocysts (E3.5) from *TopBP1* ^ki/+^ intercrosses for PCR genotyping: 21.7%, close to the expected Mendelian ratio, of embryos were *TopBP1* ^ki/ki^ homozygotes (Table 1). Although morphology was normal at isolation, all *TopBP1* ^ki/ki^ blastocysts failed to outgrow *in vitro* cultures (Fig. 2B, C). These data indicate that the TopBP1 AAD function is required for embryo development beyond the blastocyst stage.

**Table 1:** Genotype distribution of offspring from TopBP1^ki/+^ breeding.

Mating	Stage	+/+	ki/+	ki/ki	Unclear	Total
TopBP1^ki/+^ (f) x TopBP1^ki/+^ (m)	p0	22	22	-	5	49
TopBP1^+/+^ (f) x TopBP1^ki/+^ (m)	P0	15	20	-	3	38
TopBP1^ki/+^ x TopBP1^ki/+^	P0	59	108	0	-	167
TopBP1^ki/+^ x TopBP1^ki/+^	E11.5	10	21	0	17*	48
TopBP1^ki/+^ x TopBP1^ki/+^	E3.5	6	12	5	-	23

Genotyping analysis of offspring derived from AAD-mutant heterozygote backcrosses (ki/+ x +/+) and intercrosses (ki/+ x ki/+). P0: postnatal day 0; +/+, wild type; ki/+, heterozygotes mutant; ki/ki, homozygotes knock-in mutant; f, female; m, male.

* : These embryos were too small to reliably define their genotype.

### TopBP1 AAD is required for cell survival and proliferation

The lethal phenotype of TopBP1-W1147R precluded the use of *TopBP1* ^ki/ki^ MEFs for direct cellular assays. However, the presence of the base change associated with the knock-in mutation (T3439C) plus a second nucleotide polymorphism (Fig. S2A) derived from the targeting construct (T3477C, a silent mutation) offered the opportunity to specifically silence the *TopBP1^+^* allele in *TopBP1* ^ki/+^ MEFs. We designed two independent shRNA expression vectors targeted against the wild type sequence, but predicted to leave the knock-in allele resistant to RNA interference. Using co-transfection with corresponding chimeric GFP-encoding reporters, these were tested individually in COS7 cells for their ability to specifically target transcripts with the wild type (GFP-wtAAD), but not the knock-in (GFP-mutAAD) TopBP1 sequences (Fig. S3). The shRNA construct targeting the T3477C point mutation, designated shTop2, efficiently targeted GFP-wtAAD but not GFP-mutAAD. We thus transferred the shRNA construct into a vector expressing GFP to create GFP-shTop2. The co-expression of GFP from the shRNA vector will allow selection for transfected cells.

**Figure 3:**
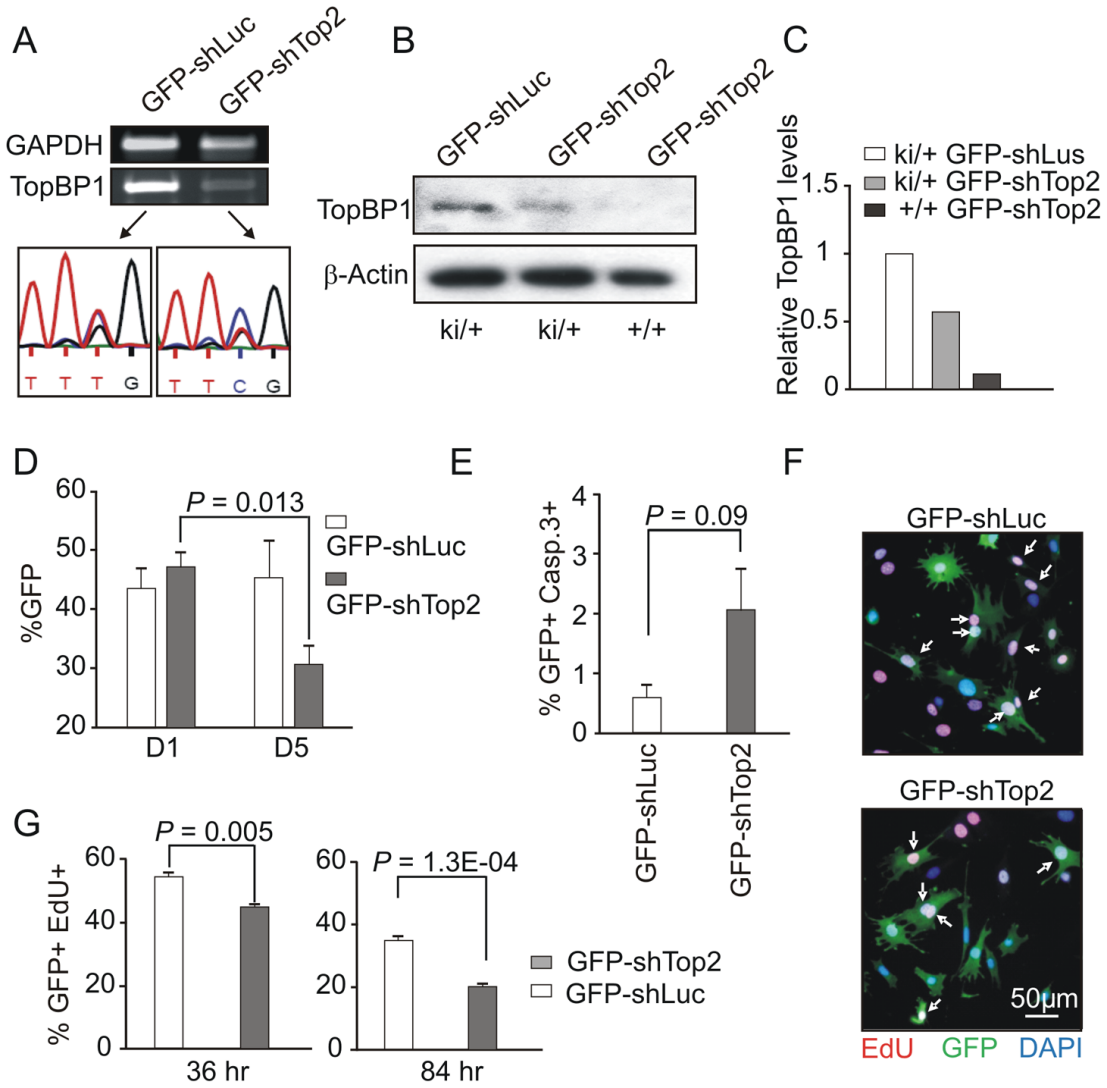
AAD mutation compromises cell proliferation and promotes cellular senescence. (**A**) RT-PCR and sequencing analysis of TopBP1 mRNA in sorted GFP-positive *TopBP1^ki/+^* cells after transfection with indicated vectors. (**B**) Immunoblot analysis of protein extracts from GFP-positive *TopBP1^ki/+^* or *TopBP1^+/+^* cells 36 hr after transfection with GFP-shLuciferase (GFP-shLuc) or GFP-shTop2. (**C**) Quantification of TopBP1 in B. Average of two independent experiments. (**D**) Percentage of GFP+ cells in the *TopBP1^ki/+^* cell population at day 1 (D1) and days 5 (D5) following shRNA transfection. Results represent 3 independent experiments for each time point. Student's *t*-test was used for statistical analysis. (**E**) Quantification of cleaved caspase 3-positive cells by immunostaining 84 hr after shRNA transfection. The data represent the mean ± SD of at least 1000 cells and two independent experiments. *P* value: Student's *t*-test. (**F**) Representative image of *TopBP1^ki/+^* cells plus-labeled with EdU (red) 36 hr after transfection with GFP-shLuc or GFP-shTop2 vectors. DNA was stained with DAPI (blue). Arrows point to EdU and GFP double positive cells (EdU+GFP+) cells. (**G**) Quantification of the percentage of Edu+GFP+ in the total GFP+ population at 36 hr and 84 hr after shRNA transfection. The data represent the mean ± SD of at least 1000 cells for each group and three independent experiments. *P* value: Student's *t*-test.

GFP-shTop2, or a GFP-shLuciferase (GFP-shLuc) control, was transfected into *TopBP1* ^ki/+^ MEFs previously immortalized by a 3T3 protocol. GFP-positive cells were sorted by FACS 36 hours after transfection (Fig. S3D). Semi-quantitative reverse transcription PCR (RT-PCR) indicated that, in comparison to GFP-shLuc control transfected cells, TopBP1 mRNA levels were reduced upon GFP-shTop2 transfection. Sequencing revealed that the remaining TopBP1 mRNA was predominantly the AAD mutant form (Fig. 3A). Western blot analysis revealed a reduction of ∼50% for the TopBP1 protein following GFP-shTop2 transfection of *TopBP1* ^ki/+^ cells when compared to GFP-shLuc control transfected cells (Fig. 3B,C). Corroboration of the specificity of GFP-shTop2 to the wild type TopBP1 transcript comes from the observation that GFP-shTop2 efficiently knocked down TopBP1 levels in *TopBP1* ^+/+^ MEF cells (Fig. 3B,C). In the following experiments, we designated GFP-shTop2-transfected *TopBP1* ^ki/+^ cells as GFP-*TopBP1* ^ki/−^ and GFP-shLuc transfected *TopBP1* ^ki/+^ cells as GFP-*TopBP1* ^ki/+^.

To identify the consequences of specific loss of TopBP1 AAD function in cell survival and proliferation we followed the proportion of the GFP positive cells after GFP-shTop2 or GFP-shLuc transfection. 24 hours after transfection, the GFP^+^ population was similar for GFP-*TopBP1* ^ki/−^ (GFP-shTop2 transfected) and control GFP-*TopBP1* ^ki/+^ cells (GFP-shLuc transfected). However, 5 days after transfection the proportion of GFP-*TopBP1* ^ki/−^ cells was significantly reduced when compared to GFP-*TopBP1* ^ki/+^ cells (Fig. 3D). Immunostaining against cleaved Caspase 3 revealed a mild (but not statistically significant) increase of apoptosis in GFP-*TopBP1* ^ki/−^ when compared to control GFP-*TopBP1* ^ki/+^ cells 4 days after of transfection (Fig. 3E). This suggests that delayed proliferation as opposed to apoptosis is the major cause of the reduced number of GFP-*TopBP1* ^ki/−^ cells. To further characterise the effect of the AAD mutation on cell proliferation, cells were pulse-labeled with EdU for 2 hours either 36 or 84 hours following transfection. Consistent with reduced proliferation, the percentage of GFP^+^ cells that were also positive for EdU in GFP-*TopBP1* ^ki/−^ cultures was reduced when compared to GFP-*TopBP1* ^ki/+^ controls (Fig. 3F, G). Nonetheless, a significant proportion of GFP-*TopBP1* ^ki/−^ cells were also EdU^+^, consistent with the AAD mutation being dispensable for DNA replication.

**Figure 4:**
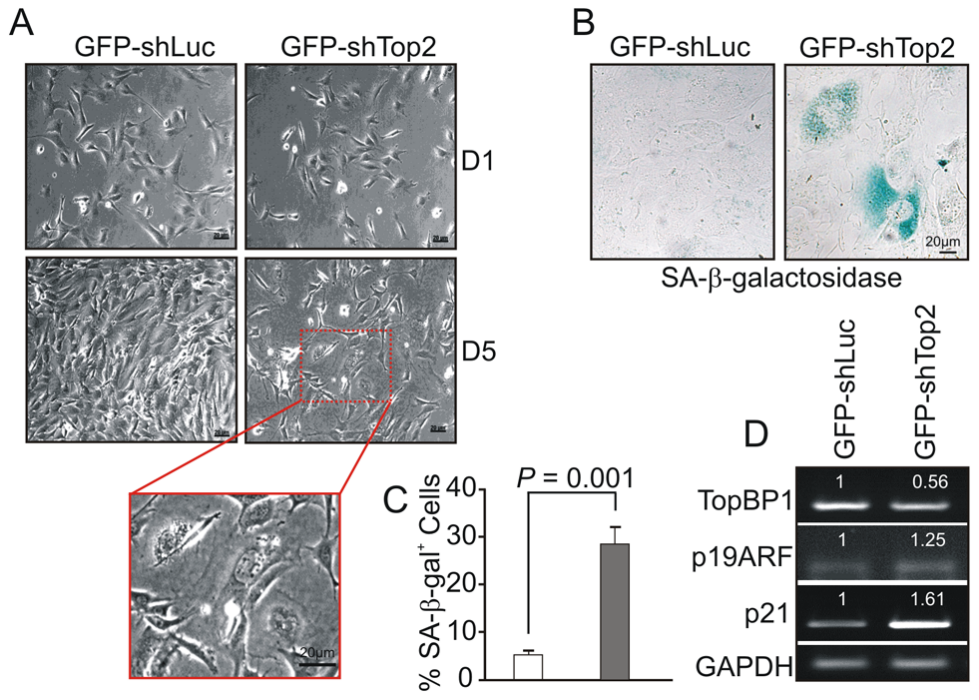
AAD mutation induces premature cellular senescence. (**A**) GFP+ *TopBP1^ki/+^* cells were sorted 24 hr after transfection and cultured. Images show the cell density and morphology at D1 and D5. Enlargement shows a representative area of *TopBP1* ^ki/−^ cells from D5. (**B**) SA-*β*-*galactosidase staining* of cells 6 days after shRNA transfection shown in blue. (**C**) Quantification of SA-β-*galactosidase positive cells* from B. The data represent the mean ± SD of at least 500 cells from 2 independent experiments. *P* value: Student's *t*-test. (**D**) Semi-quantitative RT-PCR analysis of RNA isolated from D5 cultures from A. The expression level (indicated on top of each sample) was estimated by quantification normalized to the level of GAPDH and then correlated with GFP-shLuc transfected cells. Two independent experiments were performed which showed equivalent results.

Following transfection with GFP-shLuc, control (GFP-*TopBP1* ^ki/+^) cells became confluent after 5 days in culture. However, the density of GFP-*TopBP1* ^ki/−^ cells following transfection with GFP-shTop2 did not significantly increase over the same period (Fig. 4A). GFP-*TopBP1* ^ki/−^ cells became giant and flat after 4 days in culture (Fig. 4A) and this was often associated with *β*-galactosidase positive staining, indicative of cellular senescence (Fig. 4B,C). Consistent with this, *RT-PCR* analysis revealed up-regulated expression of p19^ARF^ and p21, known senescence markers, in senescent GFP-*TopBP1* ^ki/−^ cells (Fig. 4D). We conclude that loss of TopBP1 AAD function results in proliferation defects and promotes entry into senescence.

### Activation of the ATR-Chk1 pathway in AAD-mutant cells

**Figure 5:**
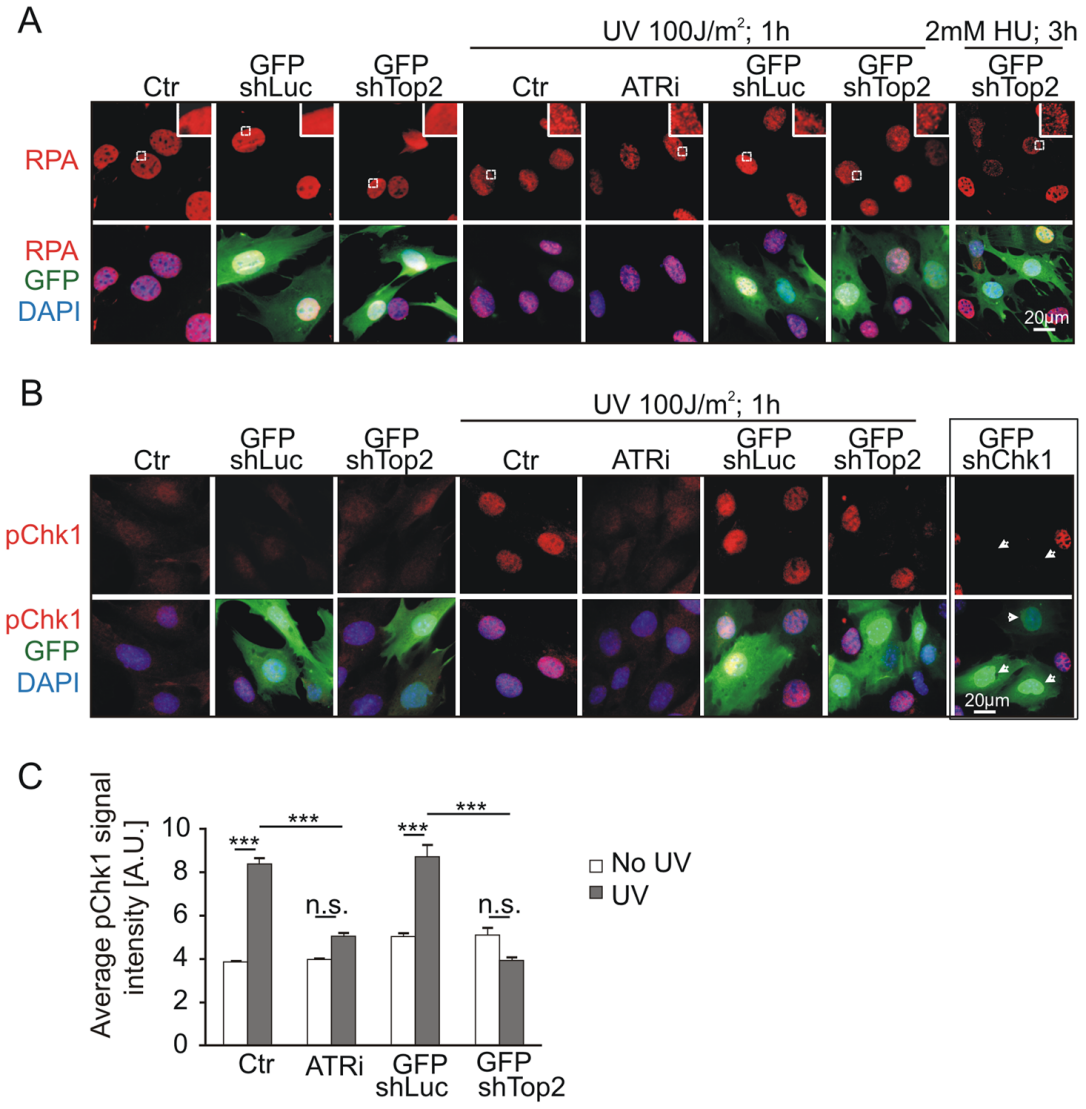
Mutation of AAD impairs ATR-Chk1 pathway *in vivo*. (**A**) Immunostaining of RPA (red and upper panel) in *TopBP1^ki/+^* cells 36 hr after transfection with GFP-shLuc or GFP-shTop2 without or with the indicated treatment. ATRi, ATR inhibitor. GFP-shLuc or GFP-shTop2 transfection is visualized by GFP (green). DNA was stained with DAPI (blue). Inset shows the enlargement of selected areas. (**B**) Immunostaining of phosphorylation of Chk1-S317 (pChk1, red, upper panel) in *TopBP1^ki/+^* cells 36 hr after transfection with GFP-shLuc or GFP-shTop2 without or with the indicated treatment. ATRi, ATR inhibitor. GFP-shLuc or GFP-shTop2 transfection is visualized by GFP (green). DNA was stained with DAPI (blue). GFP-shChk1 transfection (right panel, arrows) served as a negative control for pChk1 staining. (**C**) Quantification of fluorescent density of phosphor Chk1-S317 staining (pChk1) of indicated samples from B. The data represent the mean ± SD of at least 200 cells (or GFP positive cells) and were repeated three times. One-way ANOVA pair-test was performed for the statistical analysis. ***P<0.001; *n.s.*, not significant.

To test if the TopBP1 AAD mutation affected activation of the ATR pathway following DNA damage treatment we analyzed Chk1 phosphorylation following UV irradiation. First we established that, in our assay, UV irradiation resulted in RPA foci formation - an event occurring upstream from, and independent of, ATR activation. As expected, UV treatment resulted in similar patterns of RPA foci in GFP-*TopBP1* ^ki/−^, GFP-*TopBP1* ^ki/+^ and non-transfected control cells. Inhibition of ATR activity using the chemical inhibitor (ATRi; ETP-46464 [43]) similarly did not affect RPA localization after UV (Fig. 5A). Conversely, Immunostaining for phosphorylated Chk1 (p-Chk1-S317) to detect substrates downstream of ATR activation showed a dramatic increase of pChk1 in untransfected and GFP-*TopBP1* ^ki/+^ (shLuc transfected) cells, whereas GFP-*TopBP1* ^ki/−^ (shTop2 transfected) and ATRi-treated *TopBP1* ^ki/+^ cells showed attenuated p-Chk1-S317 staining to similar levels (Fig. 5B,C). Verifying the specificity of the assay, UV-induced p-Chk1-S317 staining was fully abrogated in *TopBP1* ^ki/+^ cells following GFP-shChk1 transfection (Fig. 5B) [44]. These data are consistent with an expectation that TopBP1 AAD function is necessary to activate ATR in response to UV treatment.

### ATR activation induced by dimerization of TopBP1 is AAD dependent

**Figure 6:**
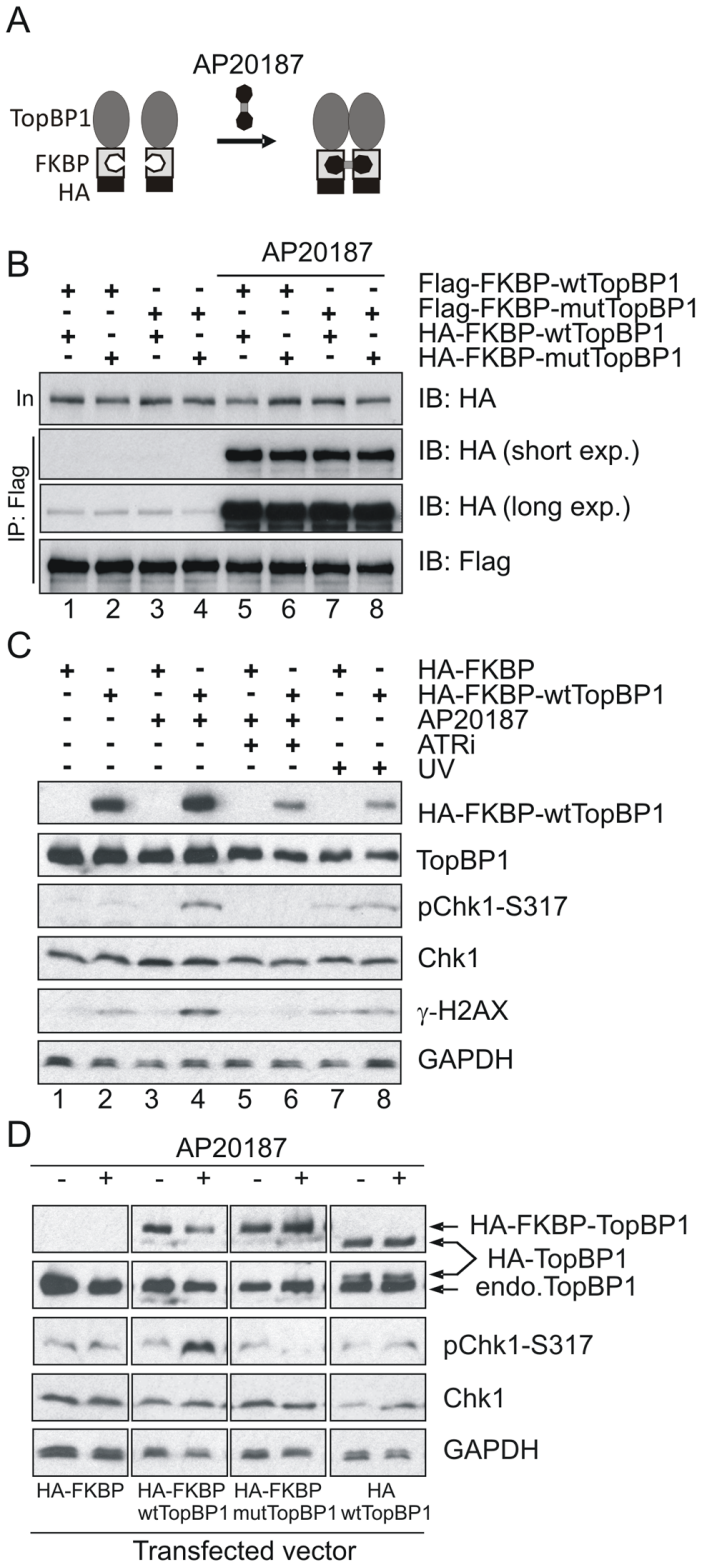
Inducible dimerization of TopBP1 activates ATR-Chk1. (**A**) Schematic showing the inducible dimerization. Addition of AP20187 induces dimerization (interaction) of FKBP-containing proteins. (**B**) Immunoprecipitation (IP) coupled immunoblot (IB) analysis of HEK293T cells that were transfected with the indicated vectors (Flag-tagged and HA-tagged). Cells were treated with 100 nm of AP20187 for 1 hr and analyzed by IP-IB using indicated antibodies. (**C**) IP-IB analysis of HEK293T cells after transfection with empty vector (HA-FKBP) or wild type TopBP1 (HA-FKBP-wtTopBP1). Cells at 40 hr after transfection were incubated with 100 nm of AP20187 for 1 hr without or with 1.6 mM of ATR inhibitor (ATRi) or were treated with 100 J/m-2 UV. Immunoblot analysis was performed 1 hr after respective treatment. (**D**) IP-IB analysis of HEK293T cells after transfection with empty vector (HA-FKBP), or FKBP-fused wild type TopBP1 (HA-FKBP-wtTopBP1), or FKBP-fused AAD mutant TopBP1 (HA-FKBP-mtTopBP1), or wild type TopBP1 without FKBP (HA-wtTopBP1). Cells were treated with 100 nm of AP20187 for 1 hour then analysed by immunoblotting with indicated antibodies.

It has previously been established that phosphorylation of human TopBP1 within the AAD at S1159 (analogous to mouse S1161) by Akt/PKB facilitates TopBP1 oligomerisation [45], [46]. To establish if our AAD mutation compromises ATR activation by preventing TopBP1 oligomerisation, we adopted an experimental approach that exploits inducible dimerization: TopBP1 was fused to FKBP-F36V (Fig. 6A), a mutant form of FKBP12 that forms a dimer upon binding to the synthetic ligand AP20187 [47]. Flag- and HA-tagged wild type TopBP1 (wtTopBP1) or TopBP1-W1147R (mutTopBP1), each fused with FKBP, were co-expressed in all combinations. Cells were then treated with AP20187 and extracts assayed for expression and co-precipitation of HA-tagged protein by the Flag-tagged protein. As expected, interactions mediated by AP2187 ligand were observed for all combinations (Fig. 6B) consistent with the expectation that a point mutation in the AAD does not disrupt FKBP-induced dimer formation.

Interestingly, when we transfected HA-FKBP-wtTopBP1 into cells we observed that the dimerization of TopBP1 induced by AP20187 promoted ATR-dependent Chk1 phosphorylation (ATRi treatment abolished phosphorylation; see lane 6). Unexpectedly, this was independent of DNA damage treatment (Fig. 6C: compare lanes 2 with 4). We next exploited this dimerisation-induced Chk1 phosphorylation to establish if the requirement for the TopBP1 AAD in ATR activation could be bypassed by forced dimerisation. HA-FKBP-wtTopBP1, HA-FKBP-mutTopBP1 and the controls HA-FKBP and HA-wtTopBP1 were each transfected into cells and AP20187 ligand-dependent Chk1 phosphorylation monitored. Neither FKBP alone or TopBP1 alone resulted in Chk1 phosphorylation in response to AP210187 ligand. As expected, HA-FKBP-wtTopBP1 expression resulted in Chk1 phosphorylation upon ligand addition (Fig. 6D). Conversely, HA-FKBP-mutTopBP1experssion did not induce Chk1 phosphorylation in response to ligand. Suggestive of a dominant negative effect, Chk1 phosphorylation in these cells was impaired below background upon AP20187-induced interaction (Fig. 6B, D). These results indicate that induced oligomerisation of TopBP1 is sufficient to induce ATR activation and subsequent Chk1 phosphorylation and that this requires the TopBP1 AAD function.

## Discussion

In both metazoans and in the yeast models, TopBP1 is required both for the initiation of DNA replication and for signalling through the ATR pathway [21]. Using the yeast models, the main function of TopBP1 in replication has been elucidated: TopBP1 acts as a scaffold, read the phosphorylation status of client proteins to promote the formation of the replicative helicase [24], [25], [26], [27]. A similar phosphorylation-dependent bridging function for TopBP1 was identified during the transmission of the ATR checkpoint [20]. An additional function for TopBP1, which was first identified in metazoans [31] and later shown to be conserved in the yeasts [11], [32], [33], is its ability to directly activate ATR through an interaction between the TopBP1 AAD domain and the ATR-ATRIP complex.

Depleting wild type TopBP1 from Xenopus and replacing this with recombinant protein in which a single aromatic residue is disrupted within the AAD abolished ATR activation in response to replication inhibitors. This suggested a key role for the AAD in activating the ATR checkpoint. However, in both the *S. cerevisiae* and the *S. pombe* model organisms, while the AAD domain of the TopBP1 homologs was similarly sufficient to activate ATR, either in vitro or in vivo, it plays a relatively minor role in ATR checkpoint signalling in response to DNA damage or replication stress. Recent data from *S. cerevisiae* identified two further ATR activating domains which play partially redundant roles in checkpoint activation: one is contained within the C-terminus of Ddc1^Rad9^, a 9-1-1 clamp subunit [48], while the other is found in the Dna2 replication protein [49]. Interestingly, compromising the function of all three AAD domains (Dpb11^TopBP1^, Ddc1^Rad9^ and Dna2) in the same cells largely abolished activation of the ATR pathway in *S. cerevisiae*. These data show that, in the yeasts, multiple ATR activation domains promote ATR activation above basal levels and that checkpoint function is dependent on ATR activation by one or more AAD domains [50].

### The TopBP1 AAD is required for embryo development

In the present study, we investigated the biological significance of the TopBP1 AAD in a mouse model. We created a single point mutation (W1147R) within the AAD of TopBP1 that removes a key aromatic residue necessary for the activation of ATR. This is predicted to separate the AAD function from other essential functions such as the scaffolding role during replication initiation and from any roles in scaffolding checkpoint complexes. Unexpectedly, this point mutation resulted in early embryonic lethality and developmental arrest at the blastocyst stage. This early lethal phenotype is equivalent to that reported for the complete knockout of TopBP1 in mice [51] and is reminiscent of the consequence of ATR deletion [52], [53]. Due to the early lethality we could not directly eliminate the possibility that the homozygous AAD knock-in (TopBP1^ki/ki^) mutation had, in fact, generated a null mutation. However, both the mRNA and TopBP1 protein levels were produced at the expected levels by the AAD knock-in allele which was visualized by specific shRNA knock-down of the wild type TopBP1 mRNA in heterozygous MEFs (TopBP1^ki/+^) (see Fig. 3B and Fig. S3B). Based on these data we propose that the TopBP1-W1147R (AAD mutant) protein is stable and the phenotypes observed are a direct consequence of the mutation introduced. Our results thus suggest that one essential function for TopBP1 in embryonic development is realized by a TopBP1 AAD-mediated ATR activation function and that this cannot be substituted for by other potential AAD domains. In addition, the scaffolding functions of TopBP1 in replication initiation and checkpoint activation cannot sustain embryonic development and are insufficient for ATR activation.

### The TopBP1 AAD plays a key role in cellular checkpoint signalling

By establishing an shRNA knock-down assay which specifically targeted the wild type, but not the TopBP1-W1147R (AAD mutant) mRNA, we were able to examine the effect of the AAD mutation in MEFs. Our first observation is that MEFs containing only mutated TopBP1-W1147R (GFP-*TopBP1* ^ki/−^) were not able to proliferate and entered senescence. This is consistent with the early embryonic lethality and strongly suggests an essential cellular role for the TopBP1 AAD, presumably by activating ATR. Our preferred explanation is that specific lesions are generated in mammalian cells during DNA replication and that, in response to these, only the TopBP1 AAD is capable of activating ATR. Such an explanation does not preclude the existence of additional ATR activating domains in other proteins (as is observed in the yeasts) but would suggest that, if these exist, they respond to alternative DNA structures or to structures formed at different points in the cell cycle, for example only in G1.

Induced DNA damage, such as that caused by UV irradiation, arrests cell proliferation via cell cycle checkpoint activation. We examined the response of cells to UV irradiation and observed that, in the absence of the wild type protein (via shRNA knock-down), cells expressing the TopBP1-W1147R (AAD mutant) protein were unable to mount a significant ATR response. The parsimonious explanation for this is that, in mammalian cells, the TopBP1 AAD is either the main or the sole mechanism for activating ATR. Given the additional complexity evident in the yeasts, this is surprising to us: evolution is prone to elaborate mechanistic pathways as organisms become multicellular and more complex. Nonetheless, our data suggest that the TopBP1 AAD is responsible for the majority of ATR signaling and that additional ATR activating domains play little or no role in metazoan checkpoint responses.

The inhibition of TopBP1 expression by antisense oligomers or by siRNA induces apoptosis in cancer cell lines or MEF cells [37], [38], [39], [40], [41]. In contrast, we did not observe a statistically significant increase in apoptosis when cells grew in the presence of the TopBP1 AAD defective protein (GFP-*TopBP1* ^ki/−^). Instead, we observed increased cellular senescence that was associated with elevated expression of p19 and p21. Full loss of TopBP1 function would be expected to disrupt replication initiation, whereas the specific loss of the AAD function may allow replication but lead to an accumulation of spontaneous damage that subsequently signals through the ATM pathway. Consistent with this, we did observe some incorporation of EdU in GFP-TopBP1^ki/−^ cells and we thus suggest that the reduced proliferation and increased cellular senescence observed in GFP-*TopBP1* ^ki/−^ cells stems from impaired G1/S transition likely resulting from ATM activation. MEF cells deleted for ATR similarly show an increase in cellular senescence, a reduction of proliferation and only a small increasing of in apoptosis [54]. This is also consistent with our expectation that the TopBP1 AAD mutation specifically affects the ATR-Chk1 cascade without preventing replication initiation.

### Forced TopBP1 oligomerisation results in ATR activation

While establishing that the AAD mutation in TopBP1 was not preventing ATR activation due to a dimerization defect, we found that forced dimerization of TopBP1 strongly stimulated ATR activation in the absence of induced DNA damage, as judged by a significant increase in Chk1phosphorylation. While oligomerisation can lead to increased protein stability and improvements to enzymatic activity [55], we did no observe any increase of the TopBP1 protein level following induced oligomerisation. Several alternative possibilities could account for ATR activation by oligomerised TopBP1: oligomerisation may enhance the affinity of TopBP1 for its interaction partners. In this regard, it is interesting to note that phosphorylation of Ser1131 (ortholog of human TopBP1 Ser1140) in the AAD of Xenopus TopBP1 enhances binding of to the Xenopus ATR-ATRIP complex, and thereby increases the capacity of TopBP1 to activate the ATR [56]. Alternatively, oligomerisation of TopBP1 may enhances its chromatin binding ability. In this regard it is interesting to note that tethering TopBP1 [57] or the *S. pombe* homolog (Rad4^TopBP1^) to chromatin [32] activates the ATR and Chk1-dependent checkpoint. As expected, despite the forced oligomerisation of the AAD mutant of TopBP1, it failed to stimulates ATR activity, strongly suggesting that TopBP1 oligomerisation is necessary but not sufficient for ATR activation and that an intact AAD is required.

## Materials and Methods

### Vector construction for gene targeting, over-expression and shRNA knockdown

Gene targeting vector was constructed with Red/ET recombineering technology (Gene Bridges). Briefly, a LoxP-Neo-LoxP cassette was inserted into bacmid (bMQ-304N19, Geneservice) encompassing the genomic region of TopBP1, using the Red/ET Quick and Easy BAC Modification Kit. The Neo cassette was subsequently excised by expression Cre recombinase in host bacterial cells, resulting in a one LoxP site in intron 20. Next, a second Flp-Neo-Flp cassette was inserted into intron 19. Mutation of Tryptophan to Arginine at 1147 was achieved by *in vivo* substitution of T3439 by C3439 (Counter-Selection BAC Modification Kit, Gene Bridges). The engineered genomic region of TopBP1 in bacmid was then subcloned into high-copy plasmid vector (ColE1) by homologous recombination, resulted in the targeting construct of TopBP1-W1147R.

Flag-tagged full length wild type or W1147R mutant TopBP1 were amplified by PCR with 5′-primer TopFL-5 (TACGGATCCCTCGGGCTCCACCTAGTTCA) and 3′- primer TopFL-3 (CCGCTCGAGGCCGTTTGACTACATTC) and constructed into pCMV-tag 2C (Stratagene), pcDNAHA, or pcDNAHA2FKBP vector, respectively [47]. GFP-tagged wild type or W1147R mutant AAD of TopBP1 were amplified by PCR with 5′-primer micTop54-2 (GAAGATCTTGACCCAGGCCTTGGAGATGAGAG) and 3′-primer micTop34-2 (ACGCGTCGACTGCCCTGGGGCTTGAGTAACACA) and constructed into pEGFP C2 (*Clontech*, Mountain View, CA, USA). The construction of shRNA expression vectors was performed as previously described [58]. Briefly, oligonucleotides targeting the coding sequences and their complementary sequences were inserted into the vector under the control of the human U6 promoter with or without CMV-driven EGFP. All the oligonucleotides contained the following hairpin loop sequence: TTCAAGAGA. The targeting sequences used were: Luciferase: GGCTTGCCAGCAACTTACA, shTop1: TGAGCAGATCATTTGGGACG, and shTop2: TGGCTTGCCAGCAACTTACA. All the constructions were confirmed by sequencing. shRNA expression vector to target Chk1 was reported as previously [44].

### Gene targeting of TopBP1 AAD mutant allele, genotyping of ES cells and mice by Southern blot, PCR and sequencing

The gene targeting vector was linearized by *Cla* I digestion and electroporated into the E14.1 ES cells. After selection with G418, correctly targeted TopBP1^W1147R^ knock-in (ki) ES clones were identified by Southern blot analysis and used to generate germline chimeric mice. To analyze the 5′-arm integration of the targeting vector into the *TopBP1* locus, ES cell DNA was digested with *Ase*I and probed with an intron-16 probe (p8) located externally to the upstream of targeting area. 3′-arm integration of the targeting vector was analyzed by digestion the DNA with *Ppu*M 1 and hybridization with an intron-27 probe (p5) located externally to the downstream of targeting area (see Fig. 1A).

For the PCR genotyping, the following primers were used: Top158: CTTCTCACTGTGCTGCTTCCTATAGC; Top159: GCTATTAATTGAGTTTTGTGAATCCC; In19-1f: GCAAGCCATGCAAGTCAATA; In19-2r: GCTTCCCCTGCTGTGATA; neo-1f: ATCTCCTGTCATCTCACCTTGC. The primer pair Top158 and Top159 was used to detect the wild type allele (wt) and targeted allele (tg) or knock-in targeted allele (ki). Combination of In19-1f, neo-1f and In19-2r detects the remove of neo-cassette in targeted allele. For sequencing genotyping of the TopBP1^W1147R^ ki allele, genomic DNAs were isolation and sequenced with primer In19-1f.

### mRNA isolation and semi-quantitative PCR

The total RNA was isolated by using Tri Reagent (T9424, Sigma-Aldrich, Munich, Germany). 1 µg of RNA was used for synthesis of first-strand cDNA by Affinity Script Multiple Temperature cDNA Synthesis Kit (200436, Stratagene) according to the manual. Semi-quantitative PCR was performed with the following primers. For TopBP1: micTop54-2 and micTop34-2 (see 4.1); for GAPDH: forward primer mGAPDH15 (GCACAGTCAAGGCCGAGAAT) and reverse primer mGAPDH13(GCCTTCTCCATGGTGGTGAA); For p19ARF: forward primer p19f (CCCACTCCAAGAGAGGGTTT) and reverse primer p19r (TCTGCACCGTAGTTGAGCAG); For p21: forward primer p21f(GTCAGGCTGGTCTGCCTCCG) and reverse primer p21r (CGGTCCCGTGGACAGTGAGCAG).

### Primary MEF isolation and cell culture, transfection, sorting and stability analysis

Primary mouse embryonic fibroblasts (MEFs) were isolated from E13.5 embryos derived from the mating between *TopBP1* ^ki/+^ mice and immortalized with a standard 3T3 protocol [59]. For transfection, 3T3 MEFs were transfected using Amaxa Nucleofector Kit R (VCA-1001, LONZA, Cologne, Germany). Briefly, MEFs were trypsinized and 1×10^6^ cells were centrifuged at 200× g for 10 min. The cell pellet was resuspended in 100 µl Nucleofector Solution mixture plus 5 g of plasmid-DNA. The cell suspension was electroporated using Nucleofector I Device (Lonza). The electroporated MEFs were cultured under normal conditions for 24 hr before FACsorting based on GFP expression. The sorted cells were either used for protein extraction, mRNA isolation or further cultured in the presence of 400 ug/ml of G418 (Invitrogen). For EdU labeling, cells were incubated with 1 µg/ml of EdU (A10044, Invitrogen) for 2 hr at 36 or 84 hr after transfection. UV exposure and HU treatment were performed at 36 hr after transfection and cells were fixed with 4% PFA for immunofluorescence staining. For ATR inhibitor treatment, 1.6 µm ATR inhibitor (ATRi) was added 1 hr before exposure to 100 J/m^2^ of UV. Cos7 or HEK293T cells were transfected with lipofectamine2000 (11668-019, Invitrogen) according the manufacturer's instruction

### Immunostaining, EdU reaction and β-galactosidase staining

Immunostaining was performed on as described previously [44]. Briefly, PFA-fixed cells were incubated with blocking buffer (1% BSA, 5% goat serum and 0.4% Triton X-100 in PBS) for 1 hr at room temperature then with a primary antibody diluted in blocking buffer at 4°C overnight followed by secondary antibodies for 2 hr at room temperature. After washing, the slides were mounted with DAPI-containing mounting medium (Invitrogen). The primary antibodies and respective dilutions are: rabbit anti-pChk1-S317 antibody (1∶100, A300-163A-3, Bethyl Laboratories, Montgomery, TX, USA); rabbit anti-Cleaved Caspase-3 (Asp175) (1∶300, 9662, Cell Signaling Technology, Danvers MA, USA) and rat anti-RPA antibody (1∶300, 2208, Cell Signaling Technology). EdU detection was carried out using a Click-iT EdU Alexa Fluor 647 Imaging Kit (953624, Invitrogen) after fixation according to the manufacturer's instruction. β-galactosidase staining was performed with a Senescence β-galactosidase staining kit (9860, Cell Signaling Technology) according to the manufacturer's instruction. Cells images were acquired using a virtual microscope (BX61VS, Olympus, Tokyo, Japan) or a confocal microscope (LSM510, Zeiss, Jena, Germany). The density of fluorescent signal was quantified by a high-content analysis microscopy (Cellomics Arrayscan VTI, Pittsburgh, PA, USA).

### Western blotting analysis

The proteins were extracted with RIPA buffer (20 mM HEPES, pH 7.6, 20% glycerol, 0.5M NaCl, 1.5 mM MgCl_2_, 0.2 mM EDTA, pH 8.0, 0.5% NP-40, 1 mM DTT, 1 mM PMSF, 5 mg/ml leupeptin, 2 mg/ml aprotinin, 1 mM β-glycerophosphate, 1 mM Na_3_VO_4_ and 10 mM NaF) from cells. After separation in SDS-PAGE, the membranes were blotted with the flowing antibodies. The primary antibodies used in this study were rabbit anti-TopBP1 antibody (1∶1000, AB3245, Millipore, Schwalbach, Germany), rabbit anti-phospho-S317-Chk1 (1∶1000, A300-163A, Bethyl Laboratories), Rabbit anti-HA (1∶10000,A190-208A, Bethyl Laboratories); mouse anti-β-Action (1∶20000, C2206, Sigma-Aldrich), mouse anti-Flag (1∶10000, F4042, Sigma-Aldrich), mouse anti- γH2AX (1∶1000, 05-636, Millipore); sheep anti-Chk1 antibody (1∶1000, ab16130, Abcam, Cambridge, UK).

### Chemically induced dimerization of TopBP1

The inducible dimerization assay was performed as previous described [47]. Briefly, HEK 293T cells were transiently transfected with pcDNAHA2-TopBP1 or pcDNAHA2FKBP-TopBP1 (or its AAD mutant counterpart). Forty hours later, transfectants were either mock-treated with 0.1% ethanol or treated with a 100 nM of the bivalent ligand AP20187 (635060, *Clontech*) and/or combined with 1.6 µm ATR inhibitor (ATRi) for 1 hr. Immunoprecipitation was carried out as previous described [47].

### Ethics statement

Animal experiments conducted in this report were approved and conducted according to the German or British animal welfare legislation and in pathogen-free conditions.

## Supporting Information

Figure S1 Structure alignment of TopBP1 AAD. Alignment of the AAD sequences from different species. The protein sequence of the AAD domain is highlighted by the solid line (under the sequence) and the sequence encoded by exon 19 and exon 20 is indicated by dashed lines (on top of the sequence). The frame marks the mouse S1147 (equivalent to W1138 in Xenopus), where a point mutation is introduced in the AAD mutant mouse model. Solid arrow points to mouse S1140 (equivalent to S1131 in humans) that is an ATM phosphorylation site. Empty arrow indicates mouse S1161 (equivalent to S1159 in humans) that can be phosphorylated by AKT. *H. sapiens*: NP_008958; *M. musculus*: NP_795953; *X. laevis*: NP_001082568; *P. troglodytes*: XP_516761; *R. norcegicus*: XP_236578; *G. gallus*: XP_418794; *C. failiaris*: XP_534266.
(PDF)

Figure S2 Expression analysis of AAD wild type and mutant TopBP1. (**A**) Sequencing results from RT-PCR products derived from +/+ and ki/+ MEFs demonstrate the introduced knock-in mutation (T3439C, W1147R), and a silent mutation (T3477C, L1159L) from the targeted allele. (**B**) Immunoblot analysis of expression of endogenous TopBP1 in *TopBP1^+/+^* and *TopBP1^ki/+^* MEF cells. Two samples of indicated genotype are shown. (**C**) Immunoblot analysis of expression of Flag-tagged wild type and AAD mutant TopBP1 in Cos7 cells. Two samples of each transfection are shown.
(PDF)

Figure S3 Establishment of AAD mutant cellular system. We took advantage of the existence of the knock-in T3439C (W1147R) mutation and of a silent mutation (T3477C) that was discovered in the sequence of the targeted allele to establish an allele-specific knock-down strategy. (**A**) Schematic of the vector-base shRNA knock-down strategy. shTop1 and shTop2 oligos are designed to specifically target wild type TopBP1 allele, but avoid the introduced mutation (T3439C, W1147R) and silent mutation (T3477C, L1159L), respectively. (**B**) A schematic diagram of screening of shRNA oligos. shRNA were transfected together with GFP-tagged wild type or mutant AAD fragment of TopBP1, respectively. GFP positive staining (green) indicates no knock-down by shRNA, whereas GFP negative cells indicate knock-down by specific shRNA. (**C**) Images of cells co-transfected shRNA and respective GFP-tagged AAD expression vectors. Control shRNA expression vector (shLuciferase, shLuc), shTop1 or shTop2 were co-transfected with GFP only, GFP-tagged wild type (GFP-wtTopBP1) or AAD mutant fragment of TopBP1 (GFP-mutTopBP1), respectively. Images were acquired 24 hr after transfection. (**D**) FACS sorting of GFP+ cells at 36 hr after transfection or knock-down as indicated. shTop2 specifically silenced the expression of GFP-wtAAD but not GFP-mutAAD.
(PDF)
