# Supplementary figures and images for: An Essential Function for the ATR-Activation-Domain (AAD) of TopBP1 in Mouse Development and Cellular Senescence
Source: PLoS Genet. 2013 Aug 8;9(8):e1003702. doi: 10.1371/journal.pgen.1003702 (PMC3738440; doi:10.1371/journal.pgen.1003702)

Zhou-Fig S1

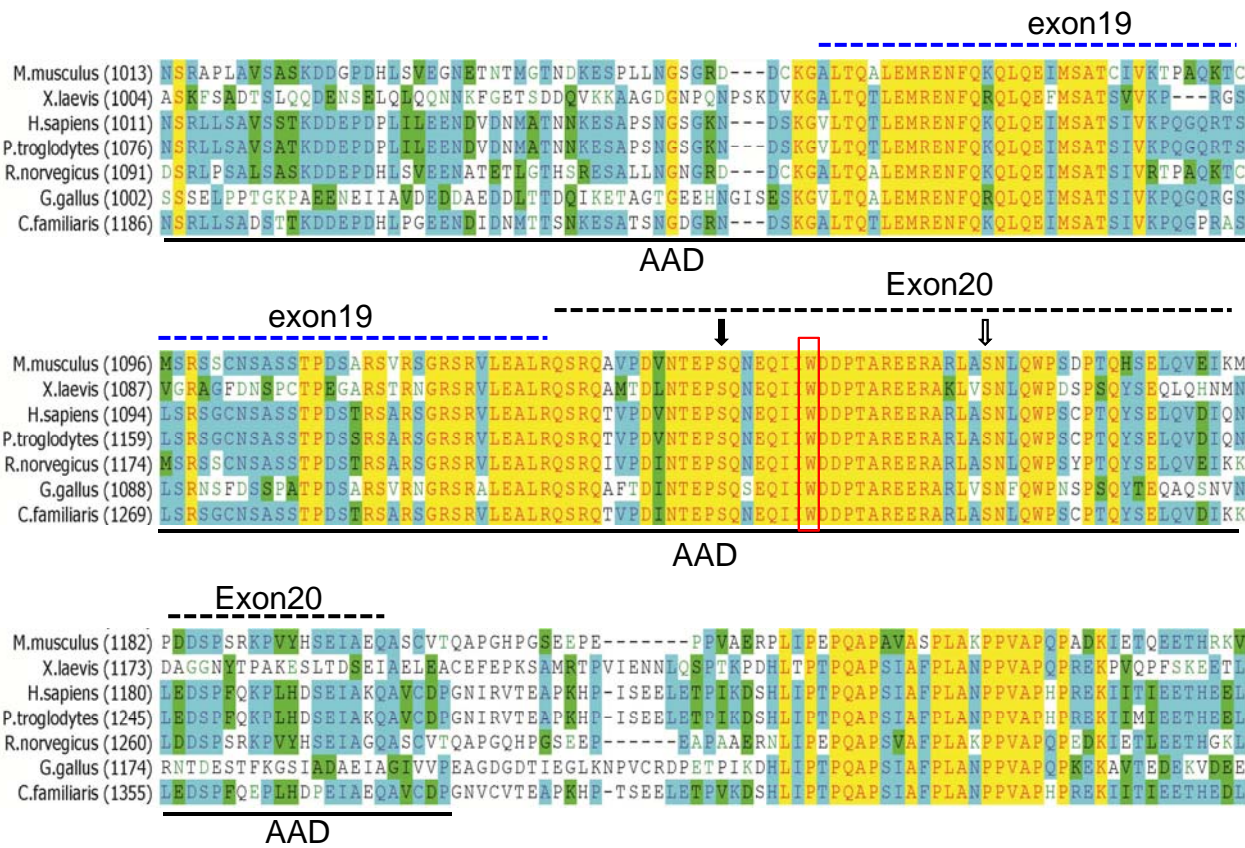

Supplement: Figure S1 — Structure alignment of TopBP1 AAD. Alignment of the AAD sequences from different species. The protein sequence of the AAD domain is highlighted by the solid line (under the sequence) and the sequence encoded by exon 19 and exon 20 is indicated by dashed lines (on top of the sequence). The frame marks the mouse S1147 (equivalent to W1138 in Xenopus), where a point mutation is introduced in the AAD mutant mouse model. Solid arrow points to mouse S1140 (equivalent to S1131 in humans) that is an ATM phosphorylation site. Empty arrow indicates mouse S1161 (equivalent to S1159 in humans) that can be phosphorylated by AKT. H. sapiens: NP_008958; M. musculus: NP_795953; X. laevis: NP_001082568; P. troglodytes: XP_516761; R. norcegicus: XP_236578; G. gallus: XP_418794; C. failiaris: XP_534266. (PDF) [file pgen.1003702.s001.pdf]

Zhou-Fig S2

A

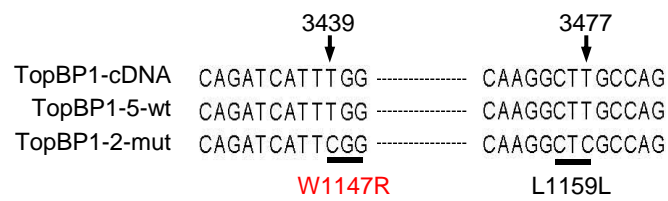

B

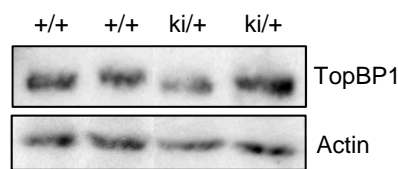

C

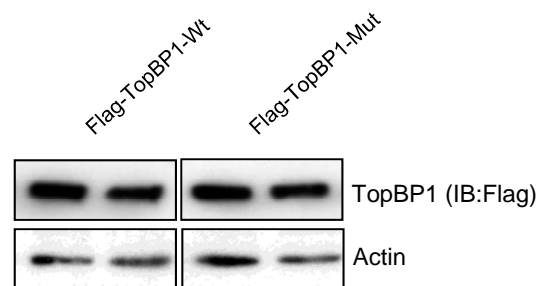

Supplement: Figure S2 — Expression analysis of AAD wild type and mutant TopBP1. (A) Sequencing results from RT-PCR products derived from +/+ and ki/+ MEFs demonstrate the introduced knock-in mutation (T3439C, W1147R), and a silent mutation (T3477C, L1159L) from the targeted allele. (B) Immunoblot analysis of expression of endogenous TopBP1 in TopBP1+/+ and TopBP1ki/+ MEF cells. Two samples of indicated genotype are shown. (C) Immunoblot analysis of expression of Flag-tagged wild type and AAD mutant TopBP1 in Cos7 cells. Two samples of each transfection are shown. (PDF) [file pgen.1003702.s002.pdf]

Zhou-Fig S3

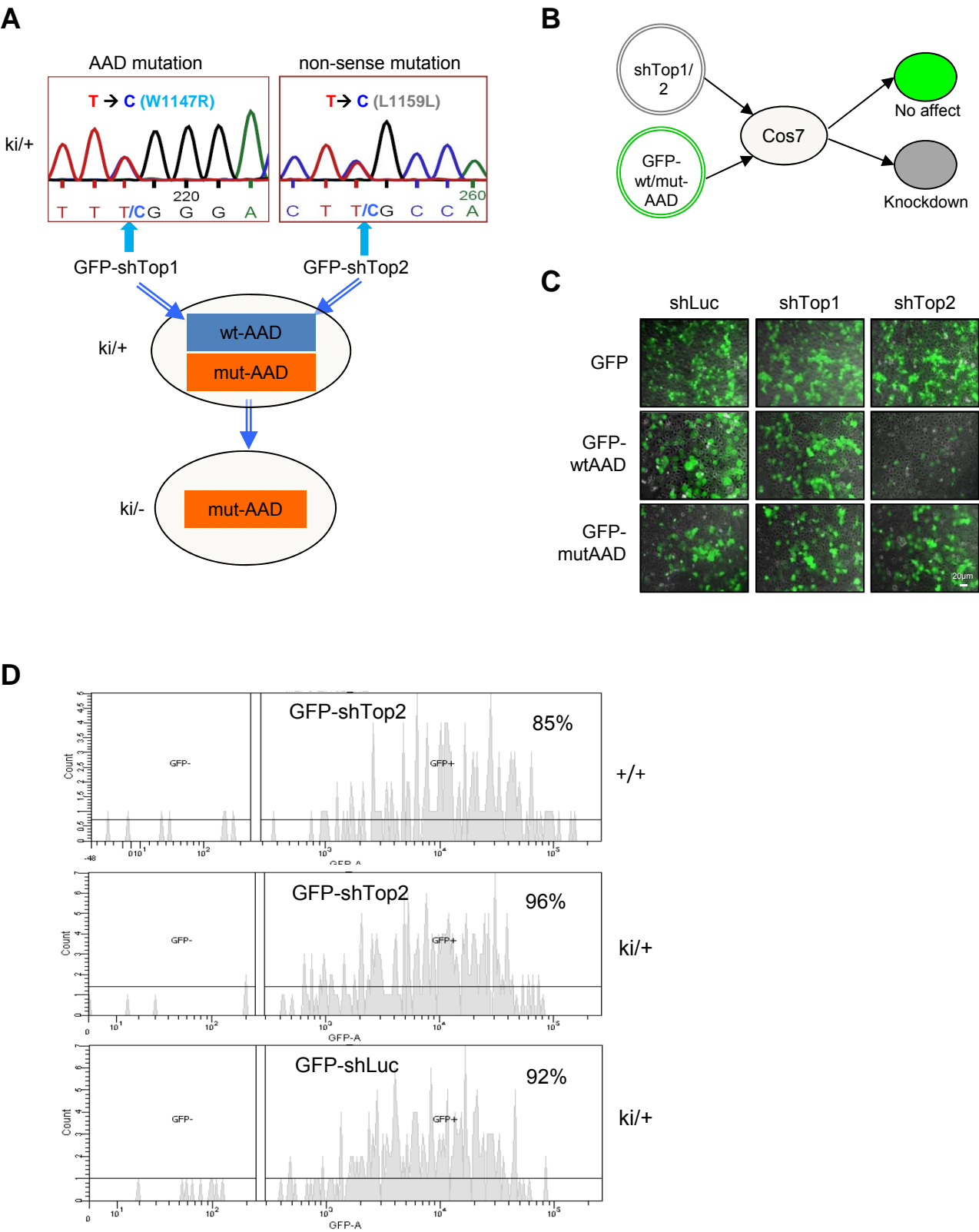

Supplement: Figure S3 — Establishment of AAD mutant cellular system. We took advantage of the existence of the knock-in T3439C (W1147R) mutation and of a silent mutation (T3477C) that was discovered in the sequence of the targeted allele to establish an allele-specific knock-down strategy. (A) Schematic of the vector-base shRNA knock-down strategy. shTop1 and shTop2 oligos are designed to specifically target wild type TopBP1 allele, but avoid the introduced mutation (T3439C, W1147R) and silent mutation (T3477C, L1159L), respectively. (B) A schematic diagram of screening of shRNA oligos. shRNA were transfected together with GFP-tagged wild type or mutant AAD fragment of TopBP1, respectively. GFP positive staining (green) indicates no knock-down by shRNA, whereas GFP negative cells indicate knock-down by specific shRNA. (C) Images of cells co-transfected shRNA and respective GFP-tagged AAD expression vectors. Control shRNA expression vector (shLuciferase, shLuc), shTop1 or shTop2 were co-transfected with GFP only, GFP-tagged wild type (GFP-wtTopBP1) or AAD mutant fragment of TopBP1 (GFP-mutTopBP1), respectively. Images were acquired 24 hr after transfection. (D) FACS sorting of GFP+ cells at 36 hr after transfection or knock-down as indicated. shTop2 specifically silenced the expression of GFP-wtAAD but not GFP-mutAAD. (PDF) [file pgen.1003702.s003.pdf]
